# The effect of treatment with pimobendan in dogs with preclinical mitral valve disease – a placebo-controlled double-blinded crossover study

**DOI:** 10.1186/s12917-021-03014-5

**Published:** 2021-09-25

**Authors:** Stephanie Klein, Ingo Nolte, Katja Rumstedt, Maximiliane Sehn, Jonathan Friedemann Raue, Franziska Weiner, Julia Sophie Treese, Martin Beyerbach, Jan-Peter Bach

**Affiliations:** 1 Clinic for Small Animals, University of Veterinary Medicine Hannover, Hannover, Germany; 2 Institute for Biometry, Epidemiology and Information Processing, WHO Collaborating Centre for Research and Training for Health at the Human-Animal-Environment Interface, University of Veterinary Medicine Hannover, Hannover, Germany

**Keywords:** Dog, Pimobendan, Mitral valve disease, Crossover, Exercise test, Biomarker

## Abstract

**Background:**

Pimobendan is a widely used medication for the treatment of dogs with congestive heart failure (CHF) and preclinical degenerative mitral valve disease (DMVD) with cardiomegaly. The benefit of a treatment in dogs with preclinical DMVD but without cardiomegaly has not yet been elucidated. Some positive effects concerning life quality and a decrease in cardiac biomarkers could be verified. This study aimed to further investigate these results using a placebo-controlled double-blinded crossover design. Out of a total of 15 dogs, eight were allocated to sequence-group AB, in which dogs received pimobendan (A) during the first treatment period and placebo (B) during the second period. Accordingly, sequence-group BA was treated first with placebo followed by pimobendan. Each treatment period lasted six months and included a baseline investigation and follow-ups after 90 and 180 days. The investigations included a questionnaire completed by the owners, echocardiographic examination, and measurements of NT-proBNP, cTnI and lactate before and after a standardised submaximal exercise test.

**Results:**

NT-proBNP values decreased significantly during the treatment period with pimobendan, and the post-exercise increase was attenuated at day 180. No significant treatment effects could be verified for cTnI and lactate, neither pre- nor post-exercise. Left ventricular size decreased under treatment, whereas no significant changes in left atrial size were detected. The owners described their dogs under treatment with pimobendan as being more active at day 90 (11/15) and day 180 (12/15). Those animals treated with placebo were described as being more active at day 90 (2/15) and day 180 (5/15).

**Conclusions:**

Pimobendan had reducing effects on the concentrations of pre- and post-exercise cardiac biomarkers and the size of the left ventricle in dogs with DMVD ACVIM B1. Exercise testing in addition to an assessment of cardiac biomarkers might improve the decision when to initiate pimobendan treatment in dogs with DMVD.

**Supplementary Information:**

The online version contains supplementary material available at 10.1186/s12917-021-03014-5.

## Background

The positive effect of treatment with pimobendan in dogs with congestive heart failure (CHF) due to degenerative mitral valve disease (DMVD) and dogs with preclinical DMVD with cardiomegaly is well established [1–4]. There is less valid information regarding the treatment of dogs with preclinical DMVD without cardiomegaly (stage B1 according to the staging system of the American College of Veterinary Internal Medicine, ACVIM) [5].

While there have been negative reports regarding the effect of pimobendan in dogs with preclinical DMVD [6, 7]*,* a recent study by our group showed some promising results concerning the effect on cardiac biomarkers and quality of life of dogs as assessed by the owners [8, 9]. In this previous study, dogs of stage ACVIM B1 were randomly allocated to either group A (receiving pimobendan) or group B (receiving placebo), and underwent echocardiography, standardised submaximal exercise testing (SSET) and investigation of cardiac biomarkers. Pre- and post-exercise values of N-terminal pro natriuretic peptide type B (NT-proBNP) were significantly lower in the pimobendan group, and a decrease in post-exercise lactate values and an increased activity perceived by the owner were noted. For the remaining examined parameters (cTnI, heart rate, echocardiographic parameters*)*, no statistically significant results could be detected. This could be due to the small patient number and variance between the patients included. Small subject numbers are a common problem in clinical trials in small animal medicine [10, 11] due to overall smaller patient numbers, less developed infrastructure for realising multi-centre studies and limited funds in comparison to human medicine. This problem is aggravated by limited owner compliance in studies including complex examinations performed over a longer period [12]. Accordingly, small animal clinical trials need to achieve the most meaningful results possible with smaller numbers of subjects compared to human medical studies.

The present study was conducted using a double-blinded, placebo-controlled crossover design to increase objectivity. To improve statistical power, the choice of best fitting statistical tests is indispensable [13]. In crossover trials, calculations of different treatment effects are based on paired samples. This allows a higher statistical power to be achieved with the same number of patients. Superior statistical power results from the possibility of within-subject comparisons, which are far less variable than between different subjects [14]. The simplest design of a crossover trial is the “two-period two-treatment” or AB/BA design [15, 16]. The intention of this design is to compare two treatments in two groups receiving a different order of treatment. This results in a so-called sequence-group, where each subject functions as its own control.

The current study aimed to further investigate the results of the predecessor study by Iwanuk et al. 2019 by implementing a crossover design, which required a more extensive study protocol but enabled an increase in statistical power. The study was designed to answer the following questions:

1. Does pimobendan have a significant effect on the tested parameters (NT-proBNP, cTnI, lactate, LVIDDn, LA/Ao, heart rate and activity) in DMVD patients in ACVIM stage B1?
2. Does pimobendan attenuate an increase in cardiac biomarkers after an SSET?
3. Are the results provided by the implemented crossover design with subsequent analysis of paired samples more robust than those provided by the analysis of unpaired samples in the predecessor study?

## Methods

### Trial design

This study was designed as a prospective, double-blinded, randomised, placebo-controlled crossover trial. It was approved by the responsible ethical review committee (Lower Saxony State Office for Consumer Protection and Food Safety, 33.9–42,502-05-14A484). All owners gave their written agreement for participation.

### Patient recruitment and examinations

Client-owned dogs with preclinical DMVD were included in the study. At admission examination, clinical history was recorded and dogs underwent clinical examination with auscultation. A characteristic left-sided systolic heart murmur had to be detected during auscultation.

During the following echocardiographic examination (Vivid E7 and E9, GE Healthcare GmbH, Solingen, Germany), mitral valve regurgitation was confirmed using color Doppler and other cardiac diseases were excluded. Dogs with minor insufficiencies of the tricuspid and pulmonary valve were not excluded from the study. Left atrial size was measured using the LA/Ao (left atrial-aortic ratio) in accordance with Hansson et al. [17]. From the right parasternal four-chamber view in long axis, measurement of the left ventricular internal diastolic diameter (LVIDD) in M-mode was performed to determine the size of the left ventricle. The parameter was corrected for body weight (LVIDDn) [18]. For inclusion in the study, at least one of the two measurements had to be within the reference values proposed in the ACVIM Consensus Statement (LA/Ao ≤ 1.6 and LVIDDn ≤1.7). Otherwise, dogs were diagnosed as having mitral valve disease in an advanced stage with cardiomegaly and were excluded from the study. Afterwards, an electrocardiogram was performed to check for possible arrhythmia.

All dogs included in the study underwent an SSET on a motorised treadmill (“quasar”, h/p/cosmos sports and medical GmbH, Nussdorf-Traunstein, Germany) as recently established by Wall et al. and utilised in the predecessor study by Iwanuk et al. [8, 9, 19]. Before starting the examinations for the study, dogs had been accustomed to the treadmill and the speed of the treadmill had been slowly increased to their respective comfort speed. This speed was then set for every follow-up investigation. The actual test was performed after a resting period and included six stages, each of which lasted three minutes. Starting at 0%, the incline was raised with every stage in steps of 4% so that the incline for stage six was 20%. After stages two and four, a three-minute break was scheduled. Between stages one, three and five, the dogs had a 20-s break. During the SSET, heart rate was recorded with a Polar® heart rate monitor (Polar FT7N and Polar H1, Polar Electro GmbH Deutschland, Büttelborn, Germany) attached to the dogs’ thorax with electrodes placed at the height of the base of the heart. The heart rate measured at rest had to increase about 40% to ensure a minimal workload, but could not exceed 240 beats per minute (bpm) as this was considered a characteristic for overstraining. In this case or other signs of overstraining, the SSET was stopped.

Immediately before and after the SSET, venous blood was collected from the Vena saphena, Vena cephalica antebrachii and in one dog from the Vena jugularis to determine pre- and post-exercise values for the biomarkers NT-proBNP, cTnI and lactate. Lactate was measured in the in-house laboratory (Cobas c 311 Analyser, Roche Diagnostics GmbH, Mannheim, Germany). EDTA-plasma and serum were processed by centrifugation at 14,000 rpm for two minutes and stored at − 80 °C until shipment to IDEXX Laboratories (Ludwigsburg, Germany). There, NT-proBNP with the Cardiopet® proBNP from the EDTA-plasma samples, and cTnI with the high-sensitive Troponin I test were measured from the serum samples. Measurements from the first blood collection included a total blood count (Adiva2120i Hematology System, Siemens Healthcare Diagnostics GmbH, Eschborn, Germany), clinical chemistry with electrolytes, and blood gases (Rapidlab 1260, Siemens Healthcare Diagnostics GmbH, Eschborn, Germany).

In addition to this, an extensive interview was conducted with the owners, and a questionnaire (see Additional file 1) with focus on changes in activity and physical endurance of the dogs was completed.

For each treatment period, follow-up investigations were performed according to the described scheme (Table 1).

**Table 1:** Examination scheme

	first treatment period			second treatment period		
	baseline	day 90	day 180	baseline	day 90	day 180
sequence-group AB	–	A	A	–	B	B
sequence-group BA	–	B	B	–	A	A

Each treatment period included follow-up investigations at day 90 and 180. A stands for the treatment with pimobendan; B stands for the treatment with placebo. Dogs in sequence-group AB received pimobendan in the first period and placebo in the second period. In sequence-group BA, the order was reversed. A washout period in between the two treatment periods was included

### Treatment protocol

Dogs were administered pimobendan and placebo tablets, which looked alike and were of similar size. The veterinarian responsible for supplying the owners with the respective medication instructed them how to administer it after every visit. The dosage was set at 0.2 mg/kg every 12 h. For better bioavailability, medication had to be administered one hour before feeding.

Dogs were randomly assigned to the sequence-groups AB or BA, with A standing for the treatment with pimobendan and B for the treatment with placebo. Hence, dogs in sequence-group AB were treated with pimobendan in the first treatment period and with placebo in the second period. The treatment periods lasted six months with a mean washout period after pimobendan treatment of 33.63 ± 13.69 weeks.

### Statistical methods

The statistical analysis was calculated with the SAS 9.4 software (SAS Institute Inc., Cary, NC, USA). Graphics were created with GraphPad Prism (GraphPad, San Diego, CA, USA).

In consideration of the visual inspection of distribution plots, a normal distribution of the studied parameters was assumed. For the statistical calculation of the treatment effects of pimobendan, analyses of variance (SAS-Procedure MIXED) for repeated measurements fitting a crossover design were used [20]. Post-hoc analysis within the framework of the repeated measures analysis of variance model was conducted using pairwise t-tests. Beyond the comparison of pimobendan and placebo treatment, changes between the examinations as well as the difference of the biomarkers before and after exercise at each day of investigation were also analysed using analyses of variance (SAS-Procedure MIXED) for repeated measurements. *P*-values less than 0.05 were considered significant. Data presented in tables and figures are indicated as mean and standard deviation (± SD) of the mean unless stated otherwise.

## Results

### Baseline characteristics

Out of a total of 22 recruited dogs, 15 were included in the final analysis. Eight dogs were allocated to sequence-group AB and seven dogs to sequence-group BA. Their baseline characteristics are presented in Table 2. Three dogs had to be removed from the study due to noncardiac diseases which required treatment with different medications during the study period. Two additional dogs progressed from ACVIM stage B1 to B2 before completing the study and were unblinded and openly treated with pimobendan. Another two participants had to be excluded due to poor owner compliance.

**Table 2:** Baseline characteristics

No.	Breed	Age	Sex	BW	HM	Group
1	Small Münsterländer	10.8	fn	19.8	I	AB
2	Golden Retriever	8.5	fn	34.5	I	BA
3	Mixed breed	11.1	mn	31.6	II	AB
4	Mixed breed	9.8	mn	16	I	AB
5	Mixed breed	7.7	fn	11.9	II	BA
6	Mixed breed	6.2	fn	6.8	I	AB
7	Jack Russell Terrier	13.5	mn	12	IV	AB
8	Jack Russell Terrier	12.4	mn	7.6	I	AB
9	Pekingese	6.2	mn	8.9	I	AB
10	Beagle	6.2	mn	18.8	IV	AB
11	Mixed breed	10.8	fn	26.6	I	BA
12	WHWT	12.9	fn	8.8	I	BA
13	Dachshund	9.9	fn	7.5	IV	BA
14	Jack Russell Terrier	15	fn	7.9	I	BA
15	Maltese	13.3	mn	8.4	III	BA

*No.* number; Breed: *WHWT* West Highland White Terrier; Age: in years; Sex: *fn* female neutered, *mn* male neutered; *BW* body weight in kilogrammes; *HM* heart murmur graded on a scale of I-VI/VI according to the Levine murmur grading scale; *Group* sequence-group

After making a calculation for each dog, trial medication dosage varied from 0.38–0.43 mg/kg/d. All dogs completed the SSET without incidents of overexertion or heart rate exceeding 240 beats per minute. However, the participants showed varying signs of physical exertion.

### Cardiac biomarkers

**Fig. 1:**
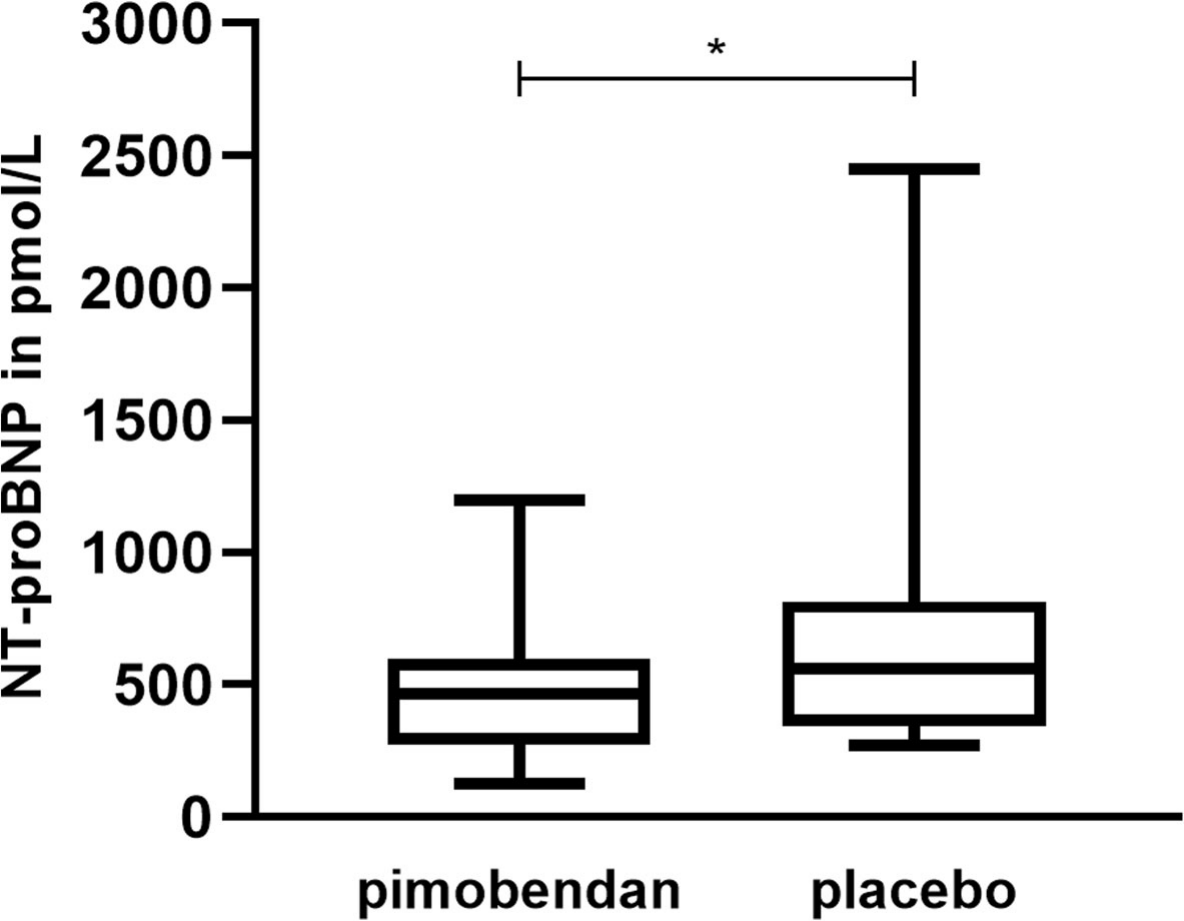
Pre-exercise NT-proBNP values at day 90. The values were significantly lower under the treatment with pimobendan than under the treatment with placebo (*p* = 0.033)

Regarding the comparison of treatment effects, baseline NT-proBNP concentrations did not differ significantly between the two treatments with pimobendan or placebo before and after exercise (Table 3). At day 90, NT-proBNP was significantly lower under the treatment with pimobendan than under the treatment with placebo before exercise (*p* = 0.033) (Fig. 1).

**Table 3:** NT-proBNP values in pmol/L

	pimobendan (A) treatment			placebo (B) treatment		
	baseline	day 90	day 180	baseline	day 90	day 180
pre-exercise	700.1 (± 401.3)	478.5 (± 291)	540.9 (± 327.1)	756.1 (± 399.7)	695.2 (± 531.1)	704.5 (± 469.9)
post-exercise	774.1 (± 368.7)	555.9 (± 300.5)	545.7 (± 371.9)	869.8 (± 492.2)	762.2 (± 592.1)	770.8 (± 521.3)
increase	74 (± 103.7)	77.4 (± 86.7)	4.7 (± 85.4)	113.7 (± 157.3)	67 (± 96.1)	66.3 (± 151.2)

NT-proBNP values for 15 dogs under the treatment with pimobendan and placebo. For the baseline- and follow-up-investigations at day 90 and 180 in each treatment period, the pre- and post-exercise NT-proBNP values are given together with their increase

**Fig. 2:**
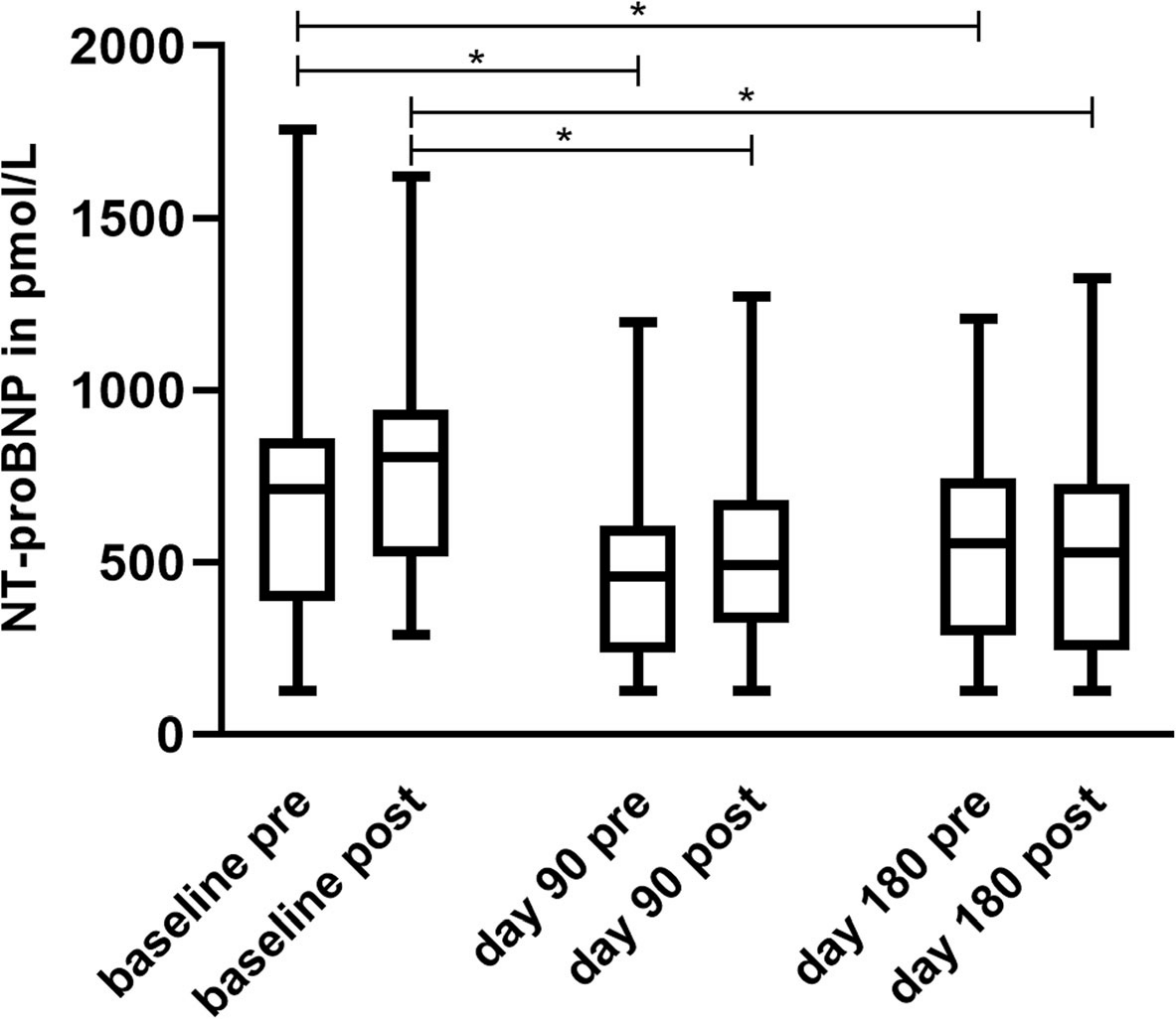
Change over time during pimobendan treatment. Pre = pre-exercise; post = post-exercise. Pre-exercise NT-proBNP values at follow-up-investigations at day 90 (*p* = 0.0021) and 180 (p = 0.0175), and post-exercise values at day 90 (*p* = 0.0028) and 180 (p = 0.0016) in the treatment period with pimobendan decreased significantly in comparison to the baseline

When regarding development over time within the two treatment periods, NT-proBNP values decreased significantly under the treatment with pimobendan from baseline to day 90 before exercise (*p* = 0.0021) and after exercise (*p* = 0.0028), and to day 180 before exercise (*p* = 0.0175) and after exercise (*p* = 0.0016) (Fig. 2).

No significant changes were detected for treatment with placebo. Baseline values did not change significantly up to day 90 before exercise (*p* = 0.4876) and after exercise (*p* = 0.2799), and up to day 180 before exercise (*p* = 0.5382) and after exercise (*p* = 0.2828).

**Fig. 3:**
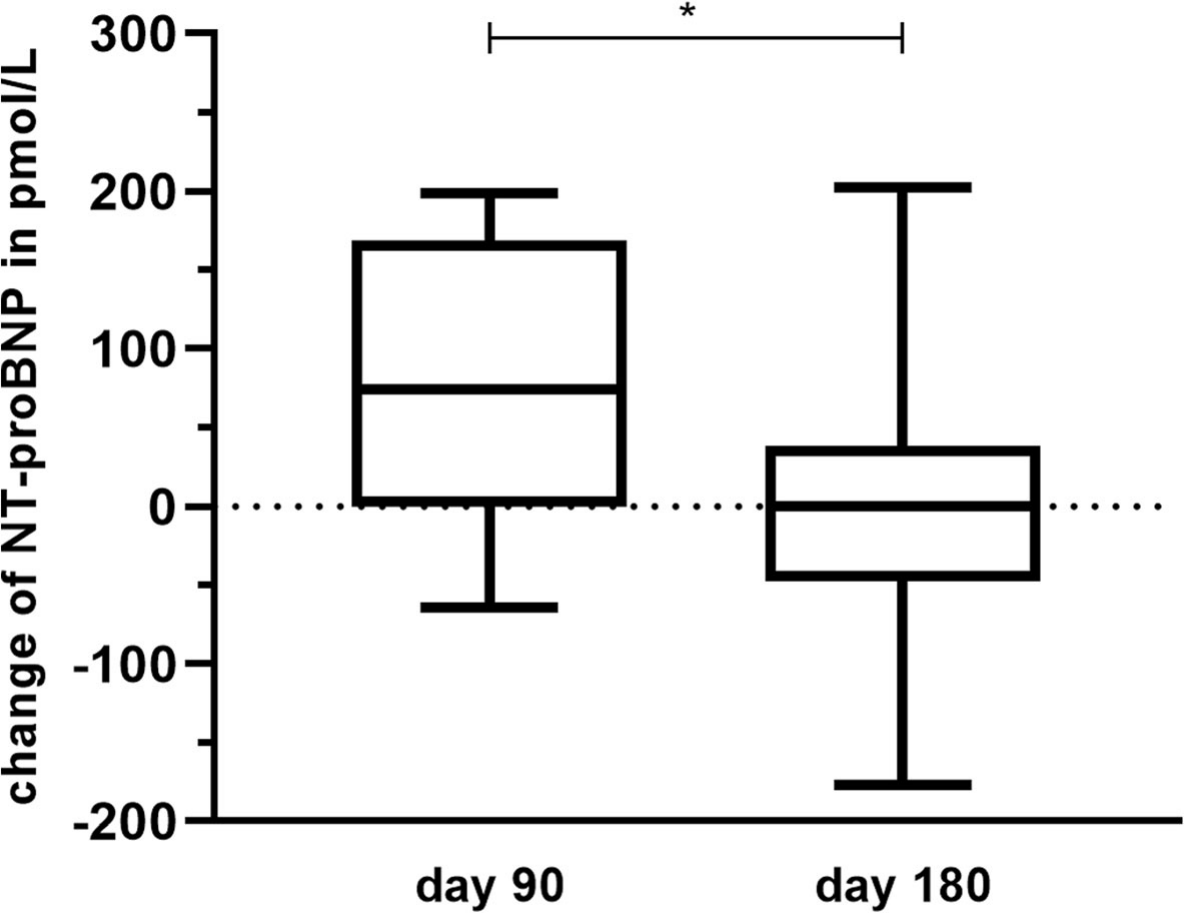
Change of NT-proBNP values under pimobendan treatment. The post-exercise increase of NT-proBNP values under treatment with pimobendan was significantly attenuated from day 90 to day 180 (*p* = 0.0452)

The increase in NT-proBNP values was significantly attenuated from day 90 to day 180 (*p* = 0.0452) under the treatment with pimobendan (Fig. 3).

**Fig. 4:**
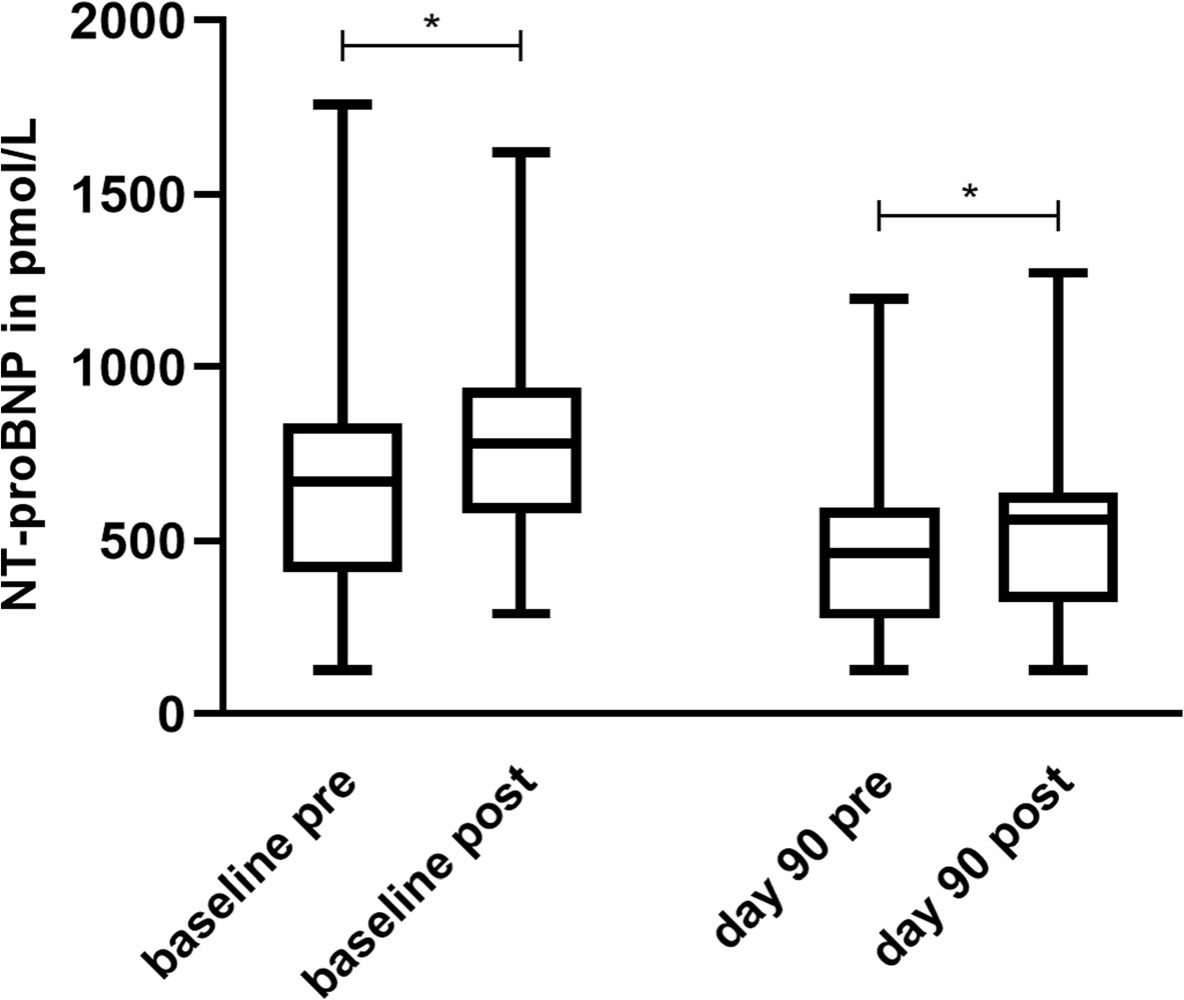
Post-exercise increase in NT-proBNP under pimobendan treatment. The post-exercise NT-proBNP values increased significantly at baseline examination (*p* = 0.0203) and at day 90 (*p* = 0.0058) under pimobendan treatment

**Fig. 5:**
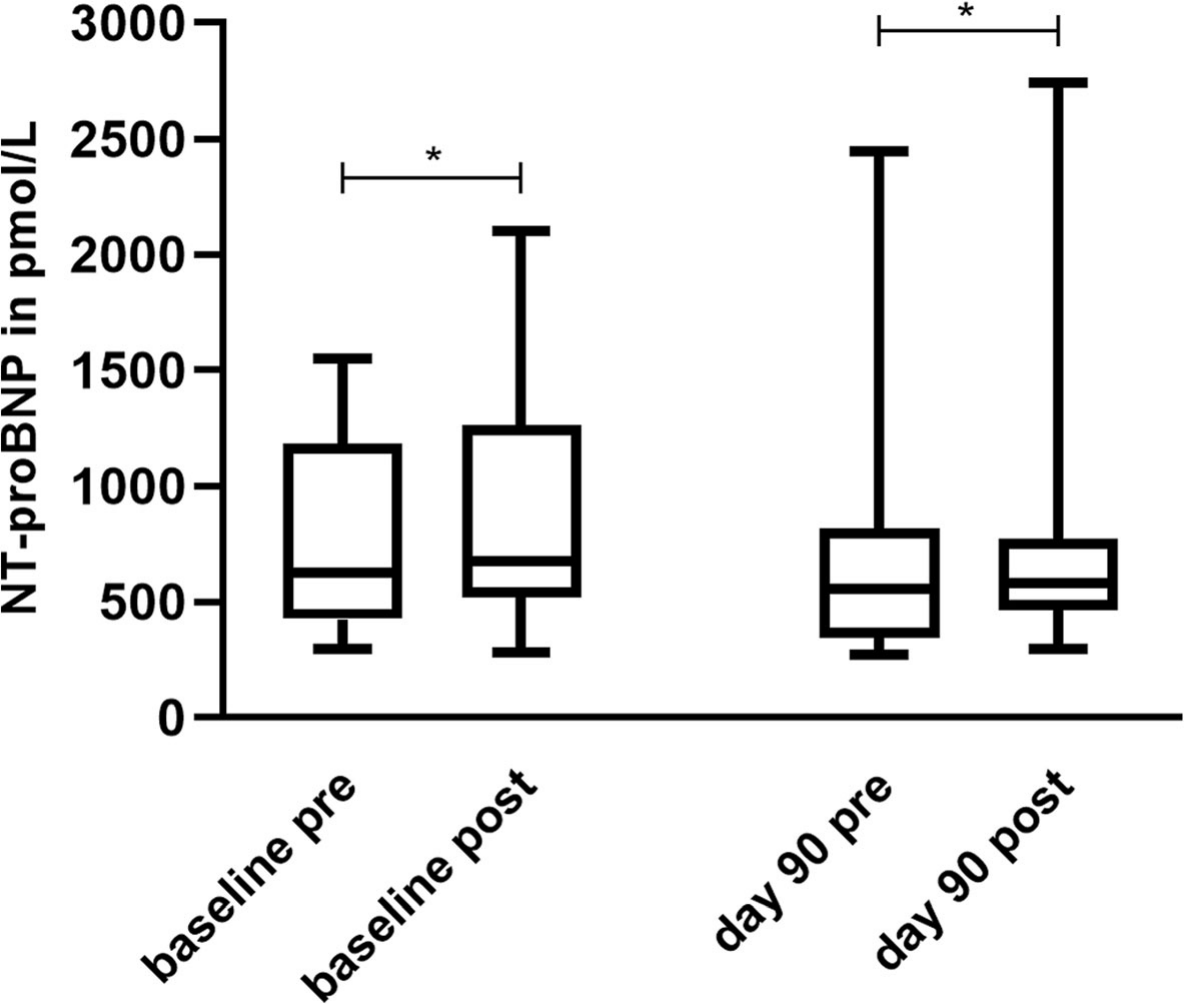
Post-exercise increase in NT-proBNP under placebo treatment. At baseline examination (*p* = 0.0142) and at day 90 (*p* = 0.0148), post-exercise values of NT-proBNP increased significantly

In both treatment periods, post-exercise values increased significantly except for day 180. The increase in NT-proBNP after exercise was significant at the baseline of the pimobendan period (*p* = 0.0203) and at day 90 (*p* = 0.0058) (Fig. 4). The increase in the placebo period was significant at the baseline (*p* = 0.0142) and at day 90 (*p* = 0.0148) (Fig. 5).

On examining cTnI, the increase after exercise was significant at the baseline of the pimobendan period (*p* = 0.0076), at day 90 (*p* = 0.0014) and at day 180 (*p* = 0.0042). The increase in the placebo period was significant at the baseline (*p* = 0.1372), at day 90 (*p* = 0.0009) and at day 180 (*p* = 0.0174). Here, no significant difference between the two treatment periods and for the entire time course could be observed.

### Echocardiography

**Fig. 6:**
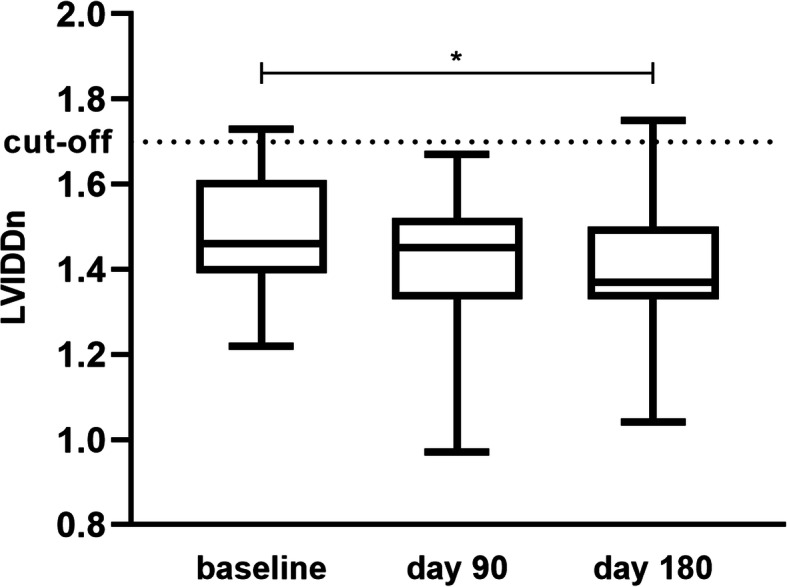
Development of left ventricle size during treatment with pimobendan. The size of the left ventricle under treatment with pimobendan decreased gradually over time with a significant difference between the baseline and day 180 (*p* = 0.032)

A significant downsizing of LVIDDn (*p* = 0.032) at day 180 under the treatment with pimobendan in contrast to its baseline value was apparent (Fig. 6). The measurements of LA/Ao and fractional shortening (FS) showed no significant differences between the two treatments (Table 4).

**Table 4:** Echocardiographic measurements

	pimobendan (A) treatment			placebo (B) treatment		
	baseline	day 90	day 180	baseline	day 90	day 180
LA/Ao	1.33 (± 0.18)	1.35 (± 0.14)	1.37 (± 0.18)	1.3 (± 0.2)	1.37 (± 0.15)	1.37 (± 0.2)
LVIDDn	1.49 (± 0.15)	1.41 (± 0.19)	1.39 (± 0.17)	1.47 (± 0.19)	1.45 (± 0.18)	1.45 (± 0.18)
FS %	35.38 (± 10.42)	39.11 (± 8.01)	35.82 (± 5.05)	33.82 (± 7.87)	34.11 (± 8.98)	32.37 (± 9.04)

*FS %* fractional shortening in per cent; Echocardiographic measurements for *n* = 15 dogs at baseline- and follow-up-investigations at day 90 and 180 in each treatment period

For the other collected data like lactate and heart rate, no significant differences were observed regardless of treatment periods or examination day.

### Questionnaire

**Fig. 7:**
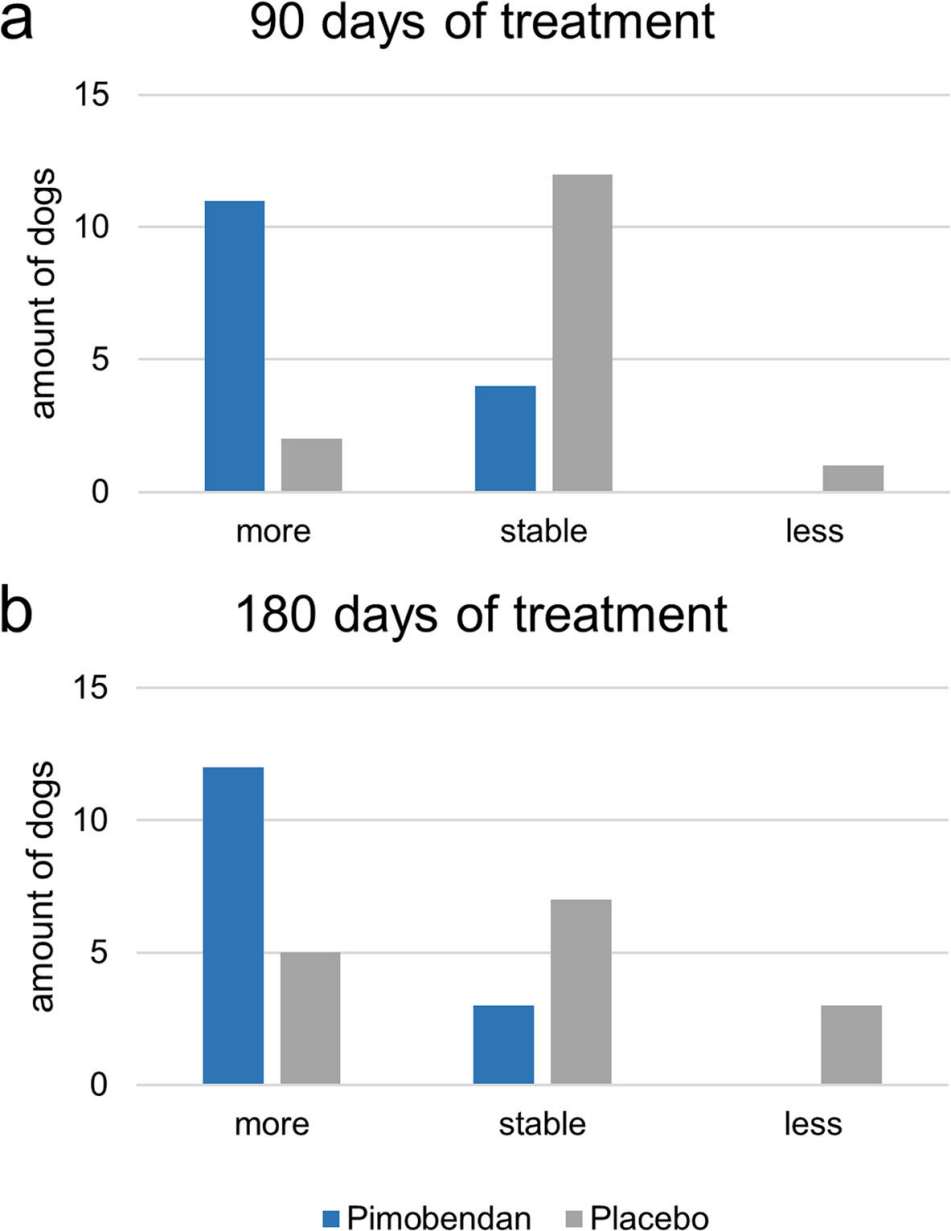
Assessment of patient activity. **A**) shows the follow-up-investigation at day 90 and **B**) shows the follow-up investigation at day 180. The blue bars represent the dogs treated with pimobendan and the grey bars the dogs treated with placebo. Change in activity could be assessed as stable and more or less active compared to baseline examination

In the questionnaire, the owners assessed their dogs’ activity at the follow-up investigations (Fig. 7). At day 90 of treatment with pimobendan, 11 out of 15 dogs were described as being more active and four dogs remained as active as before treatment. For the dogs treated with placebo, two dogs were described as being more active, whereas 12 animals remained at the baseline level and one dog was described as being less active. At day 180, the activity was evaluated as follows: for those dogs treated with pimobendan, 12 out of 15 animals were described as being more active and three animals showed no improvement. For those dogs treated with placebo, five animals were described as being more active, seven remained at the baseline level and three dogs were described as being less active.

## Discussion

Included in the answer to question number one, whether pimobendan has a positive effect on the collected data, cardiac biomarkers were the most sensitive diagnostic tools mentioned as reflecting the influence and impact of treatment. At day 90 of treatment with pimobendan, significantly lower pre-exercise NT-proBNP concentrations were apparent, which agrees with the findings of previous studies [8, 21]. The main stimulus for secretion of natriuretic peptides is increased wall stress of the left ventricle [22]. As circulating NT-proBNP is positively correlated with the amount of myocardial stretch [23], the decrease in NT-proBNP values indicates a mitigation of wall stress. This presumably results from reduced filling pressure, which is caused by a reduced pre- and afterload due to the inotropic and vasodilative effects of pimobendan [24]. Furthermore, the effect was absent at long-term follow-up at day 180, which again is in accordance with the aforementioned studies [8, 21]. Weekly variability in NT-proBNP plasma values within individuals could be a possible explanation for the lack of significant long-term results [25].

The second observed treatment effect is the significant reduction in left ventricular size measured by LVIDDn detected at day 180 of the pimobendan treatment period. Due to the previously mentioned pre- and afterload decreasing abilities of pimobendan, the left ventricular myocardium is inhibited in its maladaptive hypertrophic response to volume overload. In the study by Kanno et al. 2007, the size of regurgitant jet tended to decrease, which could lead to a reduction in the left ventricle diameter [26].

NT-proBNP and LVIDDn are independent predictors of negative outcome in dogs with DMVD [27]. The decrease in the left ventricle size might be connected with a prolonged preclinical period as it was in the EPIC study [4]. A reduction in heart size in combination with a better outcome in Doberman pinschers treated with pimobendan in the preclinical period of dilated cardiomyopathy has also been reported [28].

When assessing the results of the questionnaire, owners perceived their dogs as more active under the treatment with pimobendan. Nonetheless, some patient owners described their dogs in the placebo period as being more active as well. Although the placebo effect is not as well established in veterinary patients as it is in human patients, it could be a possible explanation for these observations. For example, a study in epileptic dogs observed a decrease in seizure frequency under the treatment with placebo [29]. In the present study, the fact that the dogs were treated and patient owners paid more attention to them might have led to an improvement in patient participation in life among the mainly older dogs. This positive interaction leads to the perception of better life quality in the animals. Improving life quality is considered to be one of the most important aspects in the treatment of chronic diseases and is even more important than survival time to patient owners [30]. These findings might indicate a positive effect of pimobendan treatment in some dogs at stage ACVIM B1. As in previous studies, no major adverse effects could be detected in the pimobendan treatment group [8, 9, 31].

The second assumption that the increase in the biomarkers after SSET would be less under the treatment with pimobendan than placebo could not be confirmed. Besides, the rise in cTnI values tended to be lower under the treatment with pimobendan. The concentrations of cTnI in the present study varied around the upper reference value of 0.06 ng/mL of the commissioned laboratory (IDEXX). They were comparable to the findings of Wall et al. 2018 for dogs at stage ACVIM B [19]. In the study by Oyama and Sisson 2004, the mean value for dogs with DMVD was 0.1 ng/mL with a cut-off value of 0.2 ng/mL for greater risk of death [32]. Plasma cTnI concentrations in the present study did not exceed this cut-off value. CTnI is cardiac-specific and indicates myocardial cell injury or necrosis. In the study by Chetboul et al. 2007, the histopathological examination revealed severe valvular lesions and signs of remodelling in asymptomatic dogs treated with pimobendan [33], whereas in the present study, the increase in post-exercise values for both treatment periods was within physiological limits, which can be referred to as normal exercise response [34–36]. Moreover, higher plasma values are correlated with age as well [37]. The comparison of NT-proBNP values between the two different treatments is subject to greater fluctuations and thus no tendency was identifiable. However, when looking solely at the pimobendan treatment period, the post-exercise rise significantly decreased from day 90 to day 180.

The NT-proBNP and cTnI results agreed with the predecessor study by Iwanuk et al. 2019. The results regarding NT-proBNP could not only be confirmed but also strengthened by a more robust statistical power of the crossover in the present study. A reducing effect of pimobendan on NT-proBNP values in consideration of between- and within-subject comparisons can be ascertained. However, significant differences for lactate values between the treatment with pimobendan and placebo could not be confirmed in the crossover calculation.

The difficulty in making a prognosis for dogs at stage ACVIM B1 is in part based on the very heterogeneous character of the preclinical period. Some dogs do not progress beyond stage B1, some develop cardiomegaly and CHF very rapidly, others show slow disease development over many years [38, 39]. In human patients, exercise testing (alone or in conjunction with measuring cardiac biomarkers) has been shown to be of prognostic value in patients with different cardiac diseases [40, 41]. Especially post-exercise BNP values increased significantly in human patients suffering from CHF or early dysfunction of the left ventricle. These were more reliable than values at rest, thus adding prognostic value [42–44]. The same might be true for canine patients. In the present study, significantly lower pre-exercise NT-proBNP values could be detected under the treatment with pimobendan and its attenuating effect during exercise at day 180. This might suggest that an exercise test with examination for sharp increases in NT-proBNP levels after exercise could be helpful in identifying dogs that are in a far advanced phase of stage B1. Further studies relating pre- and post-exercise cardiac biomarker values to progression of DMVD and survival time are necessary to confirm this hypothesis. In particular, larger cohorts or multi-centre studies including the comparison of the different disease stages with and without medication according to current therapy recommendations are needed.

Additionally, there is quite a high variability in the echocardiographic values used for the treatment decision in dogs with DMVD, with some healthy dogs exceeding the cut-off values for treatment [45]. Due to this, additional parameters for finding the optimal time-point for initiating treatment are needed. In accordance with human medicine, where post-exercise levels of cardiac biomarkers are of superior value than resting values in asymptomatic patients [44, 46], cardiac biomarkers could be used as a directional tool for decision-making for dogs.

The background to extending the predecessor study to a crossover design was to gain additional results and to confirm and strengthen the former ones with more statistical power. It was possible to confirm the results of the previous study and to additionally detect a significant reduction in the size of the left ventricle with the crossover design. Indeed, a crossover design is a valuable tool because it represents effects of medication in individual subjects and the results do not invoke the differences between different group compositions.

Nevertheless, this study has some limitations. Although a crossover design is a robust method with high statistical power, it must be taken into account that the number of subjects in the present study was rather low. Due to the long and intense study protocol, some patients were lost to follow-up examination and could not be included in the final analysis. No long-term outcome was considered to conclusively assess the effect of pimobendan on disease progression. The onset of clinical symptoms of CHF and survival time would be of outstanding importance for the final evaluation of pimobendan therapy in dogs with early mitral valve disease and need to be evaluated in further studies.

In conclusion, pimobendan has reducing effects on the concentrations of pre- and post-exercise cardiac biomarkers and on the size of the left ventricle in dogs with DMVD in stage ACVIM B1. In addition to this, most owners described their dogs as being more active under pimobendan treatment. Exercise testing in addition to assessment of cardiac biomarkers might help in making the decision when to initiate treatment in dogs with DMVD. Nevertheless, further studies investigating the effect of treatment on the progression of the disease and survival time are needed. The crossover design is a well-suited approach for comparing treatment effects. Furthermore, its implementation in the present study succeeded in gaining additional results regarding a significant reduction in the size of the left ventricle and confirming those of the predecessor study by Iwanuk et al. 2019.

## Supplementary Information

**Additional file 1.** Questionnaire.

## Data Availability

The datasets used and analysed during the current study are available from the corresponding author on reasonable request.

## Abbreviations

ACVIM: American College of Veterinary Internal Medicine
bpm: beats per minute
BW: body weight
CHF: congestive heart failure
cTnI: cardiac Troponin I
DMVD: degenerative mitral valve disease
fn: female neutered
FS: fractional shortening
HM: heart murmur
LA/Ao: ratio left atrium to aortic root
LVIDDn: left ventricular internal diastolic diameter normalised
mn: male neutered
NT-proBNP: N-terminal pro natriuretic peptide type B
pre: pre-exercise
post: post-exercise
rpm: rounds per minute
SSET: standardised submaximal exercise test
WHWT: West Highland White Terrier
