# Supplementary material for: The effect of treatment with pimobendan in dogs with preclinical mitral valve disease – a placebo-controlled double-blinded crossover study
Source: BMC Vet Res. 2021 Sep 25;17:310. doi: 10.1186/s12917-021-03014-5 (PMC8467240; doi:10.1186/s12917-021-03014-5)
Supplement: Supplementary file 1 — Additional file 1. Questionnaire. [file 12917_2021_3014_MOESM1_ESM.docx]

**Additional file**

**Questionnaire**

| **food** |  | | | | | | | | | |
| --- | --- | --- | --- | --- | --- | --- | --- | --- | --- | --- |
| **type** | O dry | O wet | | | O dry + wet | | O home-made  which?________________ __________________________________________ | | | |
| **frequency** | O once a day | | | O twice a day | | | | O three times a day | | |
| **changes during the last three months** |  | | | | | | | | | |
| **treats** | which? ___________________________________________________  how often?________________________________________________ | | | | | | | | | |
| **changes during the last three months** |  | | | | | | | | | |
| **urination** |  | | | | | | | | | |
| **frequency** | O twice a day | O three times a day | | | O four times a day | | O five times a day or more | | | |
| **dripping** | O yes | | | | | O no | | | | |
| **changes in the last three months** |  | | | | | | | | | |
| **defecation** |  | | | | | | | | | |
| **frequency** | O once a day | | O twice a day | | | O three times a day | | | O four times a day or more | |
| **quality** | O soft | | | O medium | | | | O hard | | |
| **changes during the last three months** |  | | | | | | | | | |
| **vomitus** | O yes | | | | | O no | | | | |
| **if yes** | how often?_______________________________________________  when? (after feeding, activity, etc.)_____________________________  more than in the past? O yes O no  color, content (food, bile, etc.)_______________________________ | | | | | | | | | |
| **changes during the last three months** |  | | | | | | | | | |
| **diarrhea** | O yes | | | | | O no | | | | |
| **if yes** | how often?________________________________________________  color____________________________________________________  O pasty O fluid O watery | | | | | | | | | |
| **changes during the last three months** |  | | | | | | | | | |
| **cough** | O yes | | | | | O no | | | | |
| **if yes** | how often?_______________________________________________  O during the night O after activity  O when excited O others____________________ | | | | | | | | | |
| **changes during the last three months** |  | | | | | | | | | |
| **pruritus** | O no | O yes where? _______________________________  since when?____________________________ | | | | | | | | |
| **seizure** | O no | O yes when?_________________________________  how often?______________________________  how long?_______________________________  how was it expressed?_____________________ | | | | | | | | |
| **basic diseases** | O no | O yes  which?  _______________________________________________________________________________________________________________________________________  since when? _________________________________________________________________________________________  therapy:  __________________________________________________________________________________________ | | | | | | | | |
| **foreign countries** | O no | O yes when?_______________________________________  where?______________________________________ | | | | | | | | |
| **vaccination** | O regularly  when? ___________________ | | | | | O just basic vaccination | | | | |
| **deworming** | O regularly  when?_______________________________________  how often?____________________________________ | | | | | | | | | O never |
| **drugs** | O no | O yes  which kind?___________________________________  since________________________________________ | | | | | | | | |
| **any other animals in the household** | O no | O yes  which kind?___________________________________  since________________________________________ | | | | | | | | |

**Additional questionnaire at follow-up appointments:**

| **during the last three months** | **highly increased** | **increased** | **constant** | **decreased** | **highly decreased** |
| --- | --- | --- | --- | --- | --- |
| **activity** | O | O | O | O | O |
| **endurance** | O | O | O | O | O |
| **appetite** | O | O | O | O | O |
| **thirst** | O | O | O | O | O |
| **diarrhea** | O | O | O | O | O |
| **vomitus** | O | O | O | O | O |
| **urine (volume)** | O | O | O | O | O |
| **cough** | O | O | O | O | O |

Were there any additional changes? If yes, what kind, when (at night, during the day…) and how often?

________________________________________________________________________________________________________________________________________________________________________________________________________________________________________________________________________________________________________
